# Alzheimer's disease (AD) in multiple sclerosis (MS): A systematic review of published cases, mechanistic links between AD and MS, and possible clinical evaluation of AD in MS

**DOI:** 10.1177/25424823251316134

**Published:** 2025-01-31

**Authors:** Ross Cottrill, Anupa Ekanayake, Cooper Grove, Senal Peiris, Nicholas Corbett, Biyar Ahmed, Will Jens, Tim Brearly, Sangam Kanekar, Paul Eslinger, Qing Yang, Prasanna Karunanayaka

**Affiliations:** 1 Department of Radiology, Penn State University College of Medicine, Hershey, PA, USA; 2 Department of Neurology, Penn State University College of Medicine, Hershey, PA, USA; 3 Department of Neurosurgery, Penn State University College of Medicine, Hershey, PA, USA

**Keywords:** Alzheimer's disease, dementia, multiple sclerosis, olfaction

## Abstract

**Background**: Alzheimer's disease (AD) and multiple sclerosis (MS) are two neurological disorders that can pose enormous burden to a person's quality of life. Due to new therapeutic advancements that significantly extend the lifespan, there may be an increased prevalence of AD in elderly MS patients. **Objective:** Building on a previous review on MS-AD coexistence, this review not only aimed to broaden the pool of literature searched, but also investigated possible mechanistic links between clinical markers for MS and AD. **Methods:** We searched for newly reported cases of coexisting MS and AD in PubMed, Clinical Key, BioMed Central, and Europe PubMed Central databases; and identified 101 new cases in addition to the previously reported 24 cases by Luczynski et al. (2019). The resulting 125 comorbid cases necessitated an evaluation of literature on the pathogenesis of MS and AD. **Results:** This review highlights many overlaps between AD and MS (for instance, the immune cell dysfunction, glymphatic dysfunction, genetics, environmental factors, and others). We critically evaluated clinical and laboratory metrics used to identify AD in MS patients (e.g., MRI, amyloid-β and tau protein identification, miRNA biomarker evaluation, cerebrospinal fluid analysis, vitamin levels, gut microbiota etc.). **Conclusions:** Future research should refine these diagnostic criteria and focus on enhancing screening and detection methods for AD in MS patients. Furthermore, one should also investigate the primary causes of the increased comorbidity between AD and MS.

## Introduction

Recent evidence indicates that multiple sclerosis (MS) patients have a higher risk for developing Alzheimer's disease (AD). This review intended to expand on a prior published study on MS-AD coexistence while highlighting potential mechanistic connections between clinical indicators for MS and AD.^1^ MS is an autoimmune disease of the central nervous system (CNS) characterized by inflammation, myelin degradation, gliosis, and weakening neuronal efficacy, leading to neuronal loss.^2^ Novel treatment options have extended the life expectancy of MS patients, resulting in increased susceptibility to age-related neurological diseases.^3,4^ For example, AD, which is characterized by accumulation of amyloid-β (Aβ) plaques and neurofibrillary tau tangles with concomitant neuronal dysfunction and brain atrophy, is the most common cause of adult dementia in the western world.^3,4^ Given the increase in MS patients who are living longer and the known association between aging and AD, investigating the relationships between MS, aging, and AD may provide further insights into the pathophysiology of both MS and AD.

Cognitive dysfunction is a prominent feature of MS, with up to 60% of patients having deficits in domains such as episodic memory and processing speed.^5–7^ Although cognitive decline is typically more severe in AD and less likely to affect domains such as processing speed in the early stages, issues persist in differentiating whether observed changes are due to MS pathology or other comorbidities such as AD. The fifth edition of the Diagnostic and Statistical Manual of Mental Disorders (DSM-5) further complicates the issue. The DSM-5 does not provide disease specific criteria for identifying MS-related neurocognitive disorders, potentially resulting in an over-pathologizing of MS-associated cognitive change.^8^ Compared to non-MS patients, it has been reported that physicians are often hesitant to diagnose neurocognitive disorders in MS patients at younger ages, even when cognitive decline and functional impairment are present.^9^ Rather, physicians likely attribute neurocognitive decline to their MS diagnosis leading to an underdiagnosis of AD in MS patients.^9^

A review by Luczynski et al. (2019) identified six articles, published between 1976 and 2017 on the possible coexistence of MS and AD.^1^ That study included multiple case series, case studies, and a self-reported survey.^1^ Through 2017, there were 24 published cases of confirmed coexisting MS and AD in PubMed [from 5 of the 6 articles previously reviewed by Luczynski et al. (2019)].^1^ An important contribution of that review was to provide a basis for further research by highlighting cases of MS-AD coexistence which was previously deemed unrelated. The same study provided the basis for this review to explore newly published cases of comorbid MS and AD.

In light of new scientific findings on MS-AD coexistence, an updated review of the Luczynski et al. (2019) work was deemed necessary^1^ along withreporting cases that were identified after 2018. Our review also focused on identifying mechanistic links that could explain a potentially increased risk of developing AD within the context of MS pathophysiology. Finally, we aimed to highlight recent clinical and laboratory techniques that can provide new avenues for developing improved diagnostic criteria for AD in MS patients.

## Methods

### Search criteria

Our extensive review included a focused search on coexisting cases of AD and MS. According to the components of the eligibility criteria of the PICO framework and also associated publications, our broad inclusion criteria were as follows: when considering population studies, we included elderly subjects over the age of 40 years with coexisting AD and MS (irrespective of their geographical location). We also focused on studies that clearly confirmed diagnoses of coexisting AD and MS through different types of antemortem or postmortem techniques that were carried out in a medical setting. We did not consider comparison groups and interventional outcomes as eligibility criteria. Our overarching goal was to identify and report on the updated number of cases of coexisting AD and MS. The publication characteristics included peer-reviewed studies published in English during the last 5 years. Our searches, however, had to be limited to the last five years due to unavoidable database search criteria restrictions.

Specifically, our review iwas designed to expand the findings of Luczynski et al. (2019) by searching a broader range of databases and also focusing on articles published since 2018.^1^ The information sources and the search strategy used in this review was as follows: Four databases, namely the PubMed, Clinical Key, BioMed Central, and Europe PubMed Central were searched and reviewed for cases with coexisting MS and AD. For each database, the following search criteria “‘Multiple sclerosis’ AND ‘Alzheimer's’” were applied to identify cases with the following inclusion criteria: (1) titles or abstracts included the term “Multiple Sclerosis”; (2) titles or abstracts included the term “Alzheimer's disease,” “dementia,” and/or other terms related to cognitive impairment and decline ; (3) reported at least one case of MS coexisting with AD with the exact number of reported cases; and (4) The format may be a case report, case series, case-control, or cohort study published within the last 5 years. We did not include studies with the following exclusion criteria: (1) AD-MS coexistence with other disease(s) or cognitive decline (Lewy-body dementia, Parkinson's, vascular dementia, etc.); (2) Non-Human Studies; and (3) Studies lacking confirmed diagnosis of MS and AD coexistence.

The search pattern “Multiple sclerosis AND Alzheimer's,” was applied to the Clinical Key database on July 14, 2023. Selected filters for search results included “Journal Articles,” “Full Text and MEDLINE,” and “Last 5 years.” The search produced 3109 results, and of these, 3 results matched the aforementioned criteria.

The search pattern “Multiple sclerosis AND Alzheimer's,” was applied in PubMed on July 17, 2023, with search criteria filtered to the last five years. 1936 results were found with no new articles, other than the ones already identified during the search of the Clinical Key.

The search pattern “‘Multiple sclerosis’ AND ‘Alzheimer's’,” was applied to BioMed Central on July 18, 2023 and 2203 articles met the criteria, but of these, no articles fit the previously mentioned criteria.

**Figure 1.**
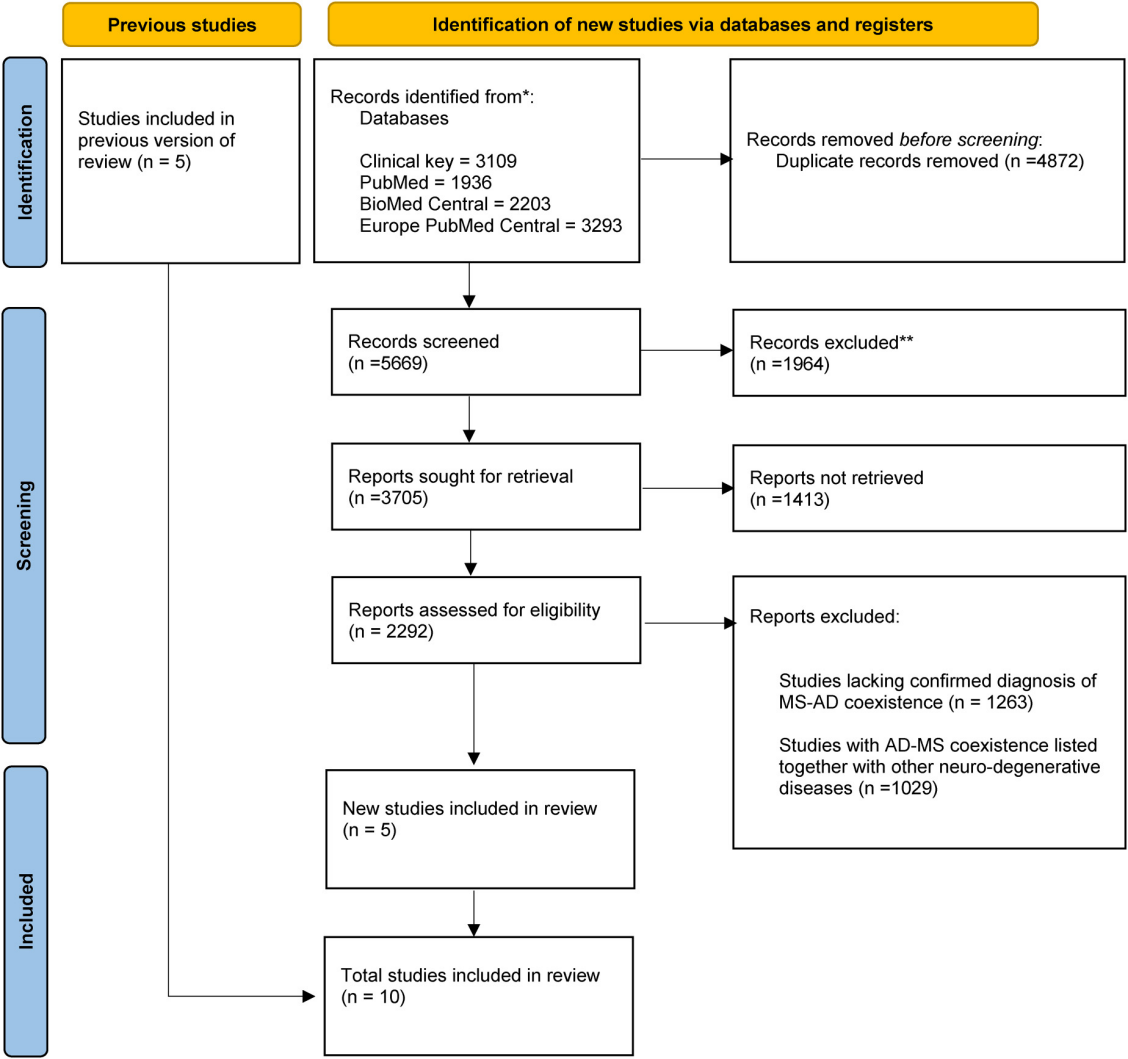
PRISMA flowchart for identifying published studies that were included in this review.

The search pattern “‘Multiple sclerosis’ AND Alzheimer's AND (FIRST_PDATE: [2018 TO 2023]) AND (((SRC: MED OR SRC: PMC OR SRC: AGR OR SRC: CBA) NOT (PUB_TYPE: “Review”)) OR SRC: PPR),” was applied to Europe PubMed Central on July 25, 2023. 3293 results were produced from the search. Four articles were identified, with two being identified in previous searches of Clinical Key and PubMed. Figure 1 show the flowchart for our search methodology.

## Results

Our initial search yielded five articles that matched the specified criteria.^10–14^ The search included 2 case reports, 2 case series, and 1 case control study. These articles identified 101 new cases in total of coexisting MS and AD reported during the last 5 years. Table 1 summarizes the findings in these studies.

**Table 1.** Summary of newly reported cases with coexisting MS and AD. Articles listed in Table 1 were the only ones that matched our inclusion criteria; the five articles identified 101 new cases of coexisting MS and AD.

Publication	Type of study	Number of cases & demography	MS subtype	MS diagnosis	AD diagnosis	Cognitive assessment	Limitations
Borrelli et al. (2020)^10^	Case report	1 female, 67 years old	PPMS-1	MRI: bilateral multiple non-enhancing periventricular demyelinating lesions and juxta cortical and infratentorial regional lesions. CSF: elevated oligoclonal bands in a type 2 pattern	^18^F-FDG PET: left side predominant temporo-parietal hypo metabolism CSF: total tau of 534 ng/L, phospho-tau of 68 ng/L amyloid-β 1–42 of 557 ng/L	Global episodic memory deficits characterized by impaired recall and recognition, severe aphasia, dyscalculia and visuo-constructive apraxia.	None shown by authors.
Londoño et al. (2022)^11^	Case series	19 Individuals, demographics not specified	RRMS-4 PPMS-3 SPMS-0 PRMS-1 CIS-2	MRI revealed typical changes of multiple sclerosis with multiple periventricular lesions, juxta cortical and infratentorial T2 hyper intensive lesions, and T1 hypo intensity.	^18^F-FDG PET: -bilateral frontal, parietal and temporal hypometabolism, including hypo metabolism in the posterior cingulate gyrus CSF-phospho-Tau concentration >61 pg./m	There were no clear discriminating features to assess multiple sclerosis-related cognitive impairment in isolation from coexistent neurodegenerative diseases.	Small sample size. Diagnostic tests were performed infrequently and only under the guidance of standard clinical therapy with no research background.
Cho et al. (2023)^12^	Case control study	78 Korean individuals of age >40 years	Not discussed	Diagnosis type not mentioned	Diagnosis type not mentioned	Not discussed	None shown by authors.
Jakimovski et al. (2020)^13^	Case report	1 female, 84 years old	PPMS-1	MRI: increase in overall T2-weighted lesion volume mild interval enlargement of the lateral ventricles.	Amyloid-based PET-increased focal uptake within the grey matter of the occipital lobe	Impairment in cognitive processing speed as well as in verbal and visual memory domains.	Lack of existing research on the cognitive abilities of older MS patients. Linear regression-based models have limitations in assessing cognitive performance due to limited aging cases and a lack of normative data for individuals over 60.
Kolanko et al. (2020)^14^	Case series	2 females over the age 60 years	RRMS-1, Other unknown	MRI: inflammatory demyelinating lesions and moderate global cerebral volume loss.	^18^F-florbetapir imaging-Positive scans had two or more brain areas of reduced or absent grey-white differentiation, or one or more areas in which grey matter activity was intense and clearly exceeded activity in adjacent white matter.	Impairments in executive function, memory (particularly encoding of new information), and processing speed.	None shown by authors.

As a complementary analysis, we searched patients with a diagnosis of MS and patients with an initial diagnosis of MS followed by a diagnosis of AD at both the national level and within the Penn State Health Network using the TriNetX database.^15^ In total, 1447 patients nationally had a diagnosis of AD followed by a diagnosis of MS. Of these, 479 patients are now deceased (see Table 2 for details). To be specific, within the last five years, 33% of patients given a diagnosis of AD in addition to MS are now deceased, whereas only 8% of patients with only an MS diagnosis are now deceased.

**Table 2.** Number of patients identified nationally and within the Penn State health network with a combination of diagnoses including MS (code: ICD-10-CM G35), AD (codes: ICD-10-CM G30, ICD-10-CM G30.0, ICD-10-CM G30.1, ICD-10-CM G30.9) after MS diagnoses, and status of “Deceased” within the last 5 years.

	Patient count	
	Penn State	US Network
1. MS patients	7310	268,643
2. MS patients + Deceased	500	21,433
3. MS patients + AD	40	1447
4. MS patients + AD + Deceased	20	479

We identified 101 new cases of comorbid MS and AD in our database searches. Adding the 24 cases^16–20^ identified by Luczynski et al. (2019), the total number of patients with MS and AD reached 125.^1^

## Discussion

### Possible mechanistic links

**Figure 2.**
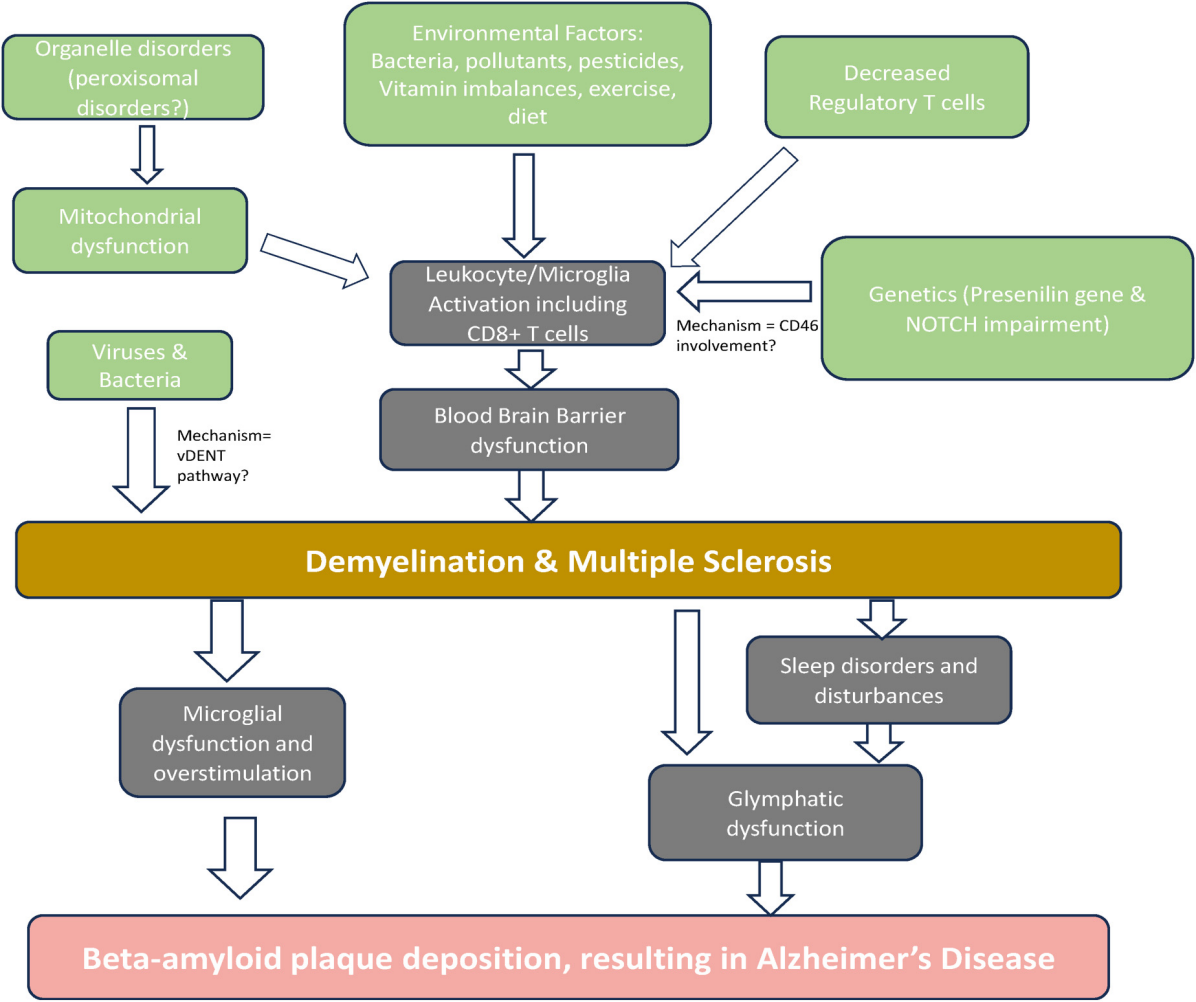
Proposed factors and their relationships contributing to the development of AD in MS patients. It is important to note that this diagram is intended to illustrate risk factors for MS and not AD. However, correlations have been observed between AD and each of the following: mitochondrial dysfunction, genetic defects, and Treg changes, which are not shown explicitly in this diagram. The focus is on risk factors for developing MS, which in turn increase the risk for developing AD. Many of these risk factors have been found to independently increase the risk for developing AD.

Since 2017, additional research, as shown in Figure 2, has been published highlighting the links between the pathogenesis of MS and AD.

The causes of MS remain uncertain and several environmental factors have been proposed. The viral demyelinating neurodegenerative trigger (vDENT) pathway has been proposed as the trigger for the first early life MS episodes that may eventually lead to AD-like cognitive impairment.^21^ James and Georgopoulos (2022) have noted correlations between AD and MS with diet, exercise, smoking, alcohol, UV radiation, vitamin D, pollutants, pesticides, viruses, and bacteria.^22^ Depp et al. (2023) conducted a study on the effect of demyelination on Aβ plaque deposition, finding that acute demyelination induced by consumption of cuprizone over a 4-week period caused significant reactive microgliosis and Aβ plaque deposition in the hippocampus.^23^ Mice bred to be congenitally deficient in myelin had more Aβ plaque deposition over their life span than healthy control mice.^23^ Based on these findings, it is reasonable to believe that there may be a causative link between demyelination and Aβ plaque deposition, directly linking the pathophysiology of MS to one of the physiological process characteristics of AD.

Overlapping genetic abnormalities leading to the dysfunction of immune system regulation may also play a role in MS-AD pathology. A genome-wide association study revealed 16 shared genetic loci between MS and AD. The genetic correlation between AD and MS, however, was non-significant, indicating that shared loci are likely related to effects with mixed direction, meaning, some are protective and others are maladaptive.^24^ It has long been established that missense mutations in presenilin proteins contribute to the development of early-onset AD.^25^ The presenilin gene is the primary catalytic unit of γ-secretases. Previous research has shown that γ-secretases create the carboxyl terminus of Aβ protein from amyloid-β protein precursors and cleave other membrane proteins such as the Notch receptor, a vital protein in cell differentiation events.^25^ Recent research has determined that the Notch pathway is likely necessary for remyelination processes, which plays a role in the regulation of immune responses involved in MS pathogenesis as well.^26,27^ Presenilin mutations, therefore, may play a part in the development of both AD and MS. However, further research is necessary to evaluate if a relationship truly exists.

Another avenue for investigating MS and AD pathogenesis has focused on peripheral immunity and the overlaps between immune responses in respective diseases. Microglia are resident cells of the CNS that play major roles in the mediation of inflammation, as well as the elimination of, protein aggregates, such as dead neurons.^28^ Microglial dysfunction and overstimulation are indicated in demyelination, creating an environment in which Aβ plaques may be able to accumulate.^23^ Peripheral immune mechanisms have also been investigated. Both MS and AD pathologies have been shown to cause peripheral leukocyte activation and further migration to the CNS.^29^ This can lead to the degradation and dysfunction of the blood brain barrier, introducing the possibility that outside cells and matter damaging to the CNS may enter. MS and AD disease courses are both improved by blocking the transmigration of T cells to the CNS (at least in some animal models), which suggests common adaptive immunity mechanisms.^29^ Regulatory T cell deficiency and dysfunction have also been found to play a role in both diseases.^29^ Stojić-Vukanić et al. (2020) investigated current literature on the role of CD8+ T cells and found that accumulation of both effector and memory CD8+ T cells were present in cognitively relevant parts of the brain in both MS and AD.^30^ Abnormal ligation of CD46 has also been noted to exist in patients with MS.^31^ Presenilin proteins have been discovered to cleave CD46 in response to infections.^31,32^ Therefore, it is possible that this immune response may be another link between presenilin and the development of MS.

Organelle dysfunction is another possible causative link between MS and AD. Mitochondrial dysfunction is a well-established similarity between MS and AD. Between this and cytokine inflammation (due to microglial cell activation), both can lead to autophagy and apoptosis. Recently, it has been proposed that peroxisomopathies may contribute to each of the aforementioned pathways.^33,34^ For example, peroxisome disorders have been hypothesized to cause mitochondrial dysfunction, oxidative stress, demyelination, and the release of proinflammatory cytokines, thus contributing to both AD and MS pathogenesis.^33^

Vitamin D is known to regulate immune system functionality and seems to play a crucial role in MS pathogenesis and progression. Vitamin D sufficiency, measured as s25(OH)D, is regarded as a contributing protective factor for MS development^35–38^ and it has been shown to influence disease severity.^39^ Gandhi et al. (2021) highlighted the role vitamin D plays in MS pathogenesis and the necessity of vitamin supplementation in MS prevention and disease management.^40^ Vitamin D deficiency has been associated with lower cognitive performance and cognitive decline, including in healthy controls. However, interventional studies with vitamin D supplementation in healthy controls and AD patients have not shown improvements in cognitive performance.^41–43^ Subsequent longitudinal studies might identify an optimal time frame in which vitamin D supplementation could protect against future cognitive decline.^41^

The pathophysiology of MS may impair the glymphatic system functionality of brain tissue, possibly increasing the risk of AD developing in MS patients. The glymphatic system removes neurotoxic excretes, such as Aβ, in AD. This clearance pathway is a highly polarized macroscopic system of convective fluid fluxes with rapid exchanges between cerebrospinal fluid (CSF) and interstitial fluid.^44^ A convective influx of CSF along the periarterial space facilitates this exchange. A combination of arterial pulsatility, slow vasomotion, respiration, and CSF pressure gradients drives CSF from the subarachnoid space into the Virchow-Robin perivascular spaces.^44,45^

Given that glymphatic function depends on cerebrospinal-interstitial fluid diffusion in the perivascular space (the region between vessel walls and astrocytic end-feet), the diffusion tensor image analysis along the perivascular space (DTI-ALPS index) can be used to measure glymphatic function indirectly.^46^ This measure has been used to investigate the relationship between brain pathology, disability, and glymphatic function in MS, with a lower DTI-ALPS index found in MS patients relative to healthy controls, as well as in progressive MS in comparison to relapsing-remitting MS phenotypes.^47^ Given the glymphatic system's role in both removing Aβ and related dysfunction in AD, the DTI-ALPS index might also aid in understanding the development of AD in MS patients.

MS pathology also produces other proteins that impart neurotoxicity on the glymphatic system. Mature astrocytes express glial fibrillary acidic protein (GFAP), which is elevated in MS patients’ plaques, suggesting astrocyte damage.^48,49^ Compared to patients with relapsing remitting MS, secondary progressive MS patients have higher CSF GFAP levels.^50^ Moreover, relapse and increased disability are linked to higher CSF GFAP levels.^51^ Patients with MS have also been found to have higher levels of nitric oxide (NO) in their serum and CSF.^52,53^ NO inhibits cytochrome C oxidase, which reduces energy production in the mitochondria, impairing their functionality.^54^ The breakdown of NO byproducts can also damage mitochondria, and there is evidence that mitochondrial dysfunction is associated with MS-associated lesions and demyelination. Additionally, by enhancing blood-brain barrier permeability, it can intensify the effects of apoptosis on neurons and glial cells and permit the entry of pro-inflammatory cells into the CNS, thus damaging brain structures including the glymphatic system.^55^

The glymphatic system is primarily active during sleep, and it is dormant during wakefulness.^56^ Due to comorbid conditions like sleep apnea, insomnia, and restless leg syndrome, the effectiveness of sleep in MS patients may be hampered. Chronic insomnia disorder may be present in up to 40% of MS patients, and MS-related symptoms such as neurogenic bladder and spasticity can be insomnia triggers.^57,58^ In addition to the usual risk factors, the neurologic dysfunction associated with MS may put patients at a higher risk for obstructive sleep apnea (OSA). In a recent study, it was discovered that MS patients had more severe OSA and central sleep apnea than matched control subjects.^59^ Given the growing body of literature showing connections between the glymphatic system, sleep, aging, depression and possible clearance and protein aggregation issues in MS and AD pathophysiology, it seems that glymphatic failures should be a primary focus of future research.^56,60,61^

### Intersections of prodromal AD and MS

In recent years, there has been a plethora of research focusing on identifying biomarkers of MS and AD. The goal is to discover new biomarkers that can identify the presence of these respective diseases earlier. The focus of investigation has ranged from cognitive and olfactory deficits to structural changes detected by neuroradiological studies, to the presence of biomarkers in bodily secretions and fluids. While many studies have identified potential biomarkers for individual diseases, there is a lack of research looking into the potential intersection between MS and AD - this is despite persistent papers supporting that there may be a link between the two. Identifying shared biomarkers of MS and prodromal AD will aid in shaping a diagnostic framework that is minimally invasive and also accurate in identifying patients with MS that require further attention for potential signs of prodromal AD.

#### Cognitive

Intersections in cognitive performance were found to be the most researched across all potential biomarkers. MS and prodromal AD are typically understood to have different cognitive profiles.^62^ AD and its prodromal state, often referred to clinically as mild neurocognitive disorder and in research settings as amnestic mild cognitive impairment (aMCI), are characterized by decline in memory retention, including both episodic and semantic memory.^63,64^ Loss of orientation and navigational deficits are also known to be associated with AD.^65^ Early in the disease course, processing speed is typically the first domain of detectable cognitive decline (although this may or may not necessarily be experienced by MS patients themselves).^63,64^ Additional domains are often affected, particularly as the disease progresses, including learning/memory and semantic fluency, suggesting a potential intersection of the cognitive profiles of patients with prodromal AD and MS.^6,62^

#### Olfaction

Olfactory dysfunction is a well-established preclinical symptom of AD, unknowingly present in most patients.^66,67^ Studies have shown correlations between certain biomarkers associated with AD such as hippocampal volume, entorhinal cortex thickness, and the presence of amyloid plaque burden on PET images leading to decreased performance on olfactory testing.^68,69^ Dan et al. (2021) suggested that there are four domains associated with a thorough investigation of olfactory function: odor discrimination, identification, threshold, and habituation.^70^ A meta-analysis conducted by Mercer et al. (2018) concluded that besides memory, smell identification is the most commonly affected domain of olfaction in AD, while olfactory sensitivity was the most commonly affected domain in elderly healthy individuals.^71^ While the connection between olfactory dysfunction and neurodegenerative diseases has been well described, investigation of the relationship between inflammatory processes associated with MS and olfactory dysfunction has only gained ground in recent years. A wide-scoping review by Mirmosayyeb et al. (2022) suggested that there is a higher prevalence of olfactory dysfunction in patients with MS when compared to healthy controls.^72^ Smell loss in MS patients is associated with increased levels of anxiety and decreased quality of life. Therefore, olfactory testing is imperative and can be beneficial for most patients with MS.^73,74^

A study by Deluca et al. (2015) found that olfactory bulb and tract demyelination were present in 70.6% of the 17 MS patients that were sampled postmortem.^75^ While this may support that there is reason for MS alone to cause olfactory dysfunction, one has to consider that postmortem studies relate to very late stages of the disease course and do not provide clinical data capable of guiding decision making in cognitively impaired MS patients. Bsteh et al. (2020) used the Burghart Sniffin’ Sticks test on a cohort of MS patients and concluded that smell threshold was associated with inflammation independent of neurodegeneration, while smell identification and discrimination were associated with the degree of neurodegeneration independent of inflammation.^76^ While it is difficult to discern whether the olfactory dysfunction in MS patients is associated with AD, one interesting point of intersection is that the loss of smell identification in both AD and MS patients is associated with prolonged disease course and neurodegeneration.^76,77^ Odor identification scores have also been shown to have a negative correlation with disease duration.^77^ Therefore, investigating olfactory function in MS patients can provide easily obtainable information that may be helpful in guiding further interventions.

Due to the apparent increased risk of developing AD in MS patients, poor diagnostic criteria, and evaluation protocols for MS patients with progressive cognitive decline, we outline below potential clinical and laboratory/imaging evaluation methods available to aid in the diagnosis of AD in patients with MS.

One area for possible future research may be risks of medications for MS. Other than the diagnosis itself, medications are one of the key differences between the MS population and other adults. While it has not been sufficiently studied, it is possible that this may contribute to the development of AD, or it could act as a protective factor. Following research in this area, it may be determined which medications should be prescribed to individuals with a diagnosis of MS, and which medications should be prescribed to MS patients who go on to develop AD.

### Clinical evaluation and reasoning

This review has demonstrated the elevated risk that MS patients have for developing AD or a relatively AD-like syndrome. Thus, there is a need for a more succinct diagnostic criteria based on easily available clinical data to identify MS patients that may be at risk for developing AD so disease modifying treatment can be started as early as possible. This review suggests that there are intersections of MS and prodromal AD in both cognitive and olfactory domains.

MS patients should undergo appropriately tailored cognitive evaluation using tests sensitive to both MS and AD, as cognitive deficits are reliably indicative of underlying pathology both shared and unique to each. MS patients with noted deficits in semantic fluency might be prioritized for further workup for prodromal AD.^6,62^

Olfactory testing provides another crucial piece of obtainable data and may even be integrated into screening or subspecialty cognitive evaluations (e.g., using established tests such as the Smell Identification Test). Testing for olfactory function should include at least the following three major domains in order to be thorough: odor identification, threshold, and discrimination. The olfactory deficit domains are different in MS patients depending on disease progression, but MS patients identified as having impaired odor identification should be further investigated for their risk of developing AD, as this is the most commonly affected domain in AD. The source of this dysfunction in AD has been indicated to be due to olfactory decline rather than memory/language decline.^76,77^ MS patients have previously been shown to have an increased risk of olfactory loss as well.^78^ However, it has not been determined to what extent this olfactory loss may differ between patients with MS versus AD, so additional research is needed to obtain definitive guidelines to differentiate olfactory loss caused by MS versus AD.

No study in this review combined different types of clinical data to identify MS patients who might be at a higher risk for AD. Further research should be conducted combining the previously stated clinical data, along with imaging and CSF biomarkers, in order to determine the sensitivity and specificity of available clinical data in identifying MS patients who are prone to develop AD.

### Laboratory and imaging evaluation and reasoning

Upon evaluation of recent literature, several methods for detecting AD may be applicable to patients with MS. Further investigations will need to be completed to determine sensitivities and specificities of these tests for the diagnosis of AD in MS patients. With those findings, more specific diagnostic criteria may be formulated. Outlined below are several avenues to differentiate AD from cognitive decline symptoms of MS using laboratory testing and/or imaging:

#### FDG and amyloid PET imaging

Both FDG-PET and Amyloid PET imaging have been verified as effective means for identifying AD. FDG-PET is able to detect a reduced brain metabolism in the neurodegeneration seen in AD. FDG-PET, while previously included as a biomarker within the ATN framework, was recently supported as an independent biomarker of AD, leading to the proposal of an ATN(F) framework.^79^ However, other studies have indicated that FDG-PET has a low specificity for AD and a lower sensitivity for AD than Amyloid PET.^80,81^

Amyloid PET imaging evaluates the deposition of amyloid pathologies present in AD using various radiopharmaceuticals such as Pittsburgh Compound B and Florbetapir (18F). It was previously determined that Amyloid PET using Florbetapir (18F) provided a sensitivity and specificity of over 92%.^82^ Evaluations of Aβ and tau biomarkers in patients with MS tend to show decreased deposition of Aβ with no significant change in tau accumulation. Following this study, it was concluded that MS may somehow slow the accumulation of Aβ.^83^ Rates of Aβ deposition have not been sufficiently studied in patients with MS who are suspected of having co-existing AD. However, an Aβ accumulation in a patient with MS that is normal or high (not the lower value typically seen in MS) may be indicative of co-existing AD. Further studies are needed to investigate if this hypothesis holds true. Kolanko et al. (2020) were able to diagnose AD in two patients with MS (reported in the ‘Results’ section of this study) using Amyloid PET.^14^

#### Amyloid-β and tau protein

Pathological changes in amyloid metabolism is a hallmark trait of AD.^84^ It has been shown that AD-related cognitive impairment usually accompanies reduced CSF Aβ_42_ levels.^85–87^ Conversely, tau protein is implicated in stabilizing functions for several cytoskeletal proteins and regulates their assembly. In AD, Tau proteins form neurofibrillary tangles that accumulate, leading to neurodegeneration in the presence of Aβ.^88,89^ Aβ_42_ and tau were found to be associated with lesion load and grey matter loss,^88^ suggesting that inflammation might alter Aβ_42_ expression and thus cause diffuse cerebral disconnection.^90–92^ As such, tau and proteins of amyloid metabolism and precursor-like proteins might be useful in monitoring the longitudinal trajectory of cognition in MS patients.^91,93,94^ Monitoring of tau proteins may be accomplished using tau PET.

#### MRI evaluation of various brain region volumes

When comparing MS to Parkinson's disease, mild cognitive impairment, AD, and healthy controls, MS had significantly lower white matter volume compared to all other disease groups. AD was the only disease found to have significantly lower gray matter and cortical volume compared to MS patients.^95^ Therefore, reduced gray matter volume and cortical volume in patients with MS may be a useful result pointing to a diagnosis of AD.

#### Evaluation of biomarkers in CSF

CSF evaluations have long been a staple in providing diagnostic biomarkers for the presence of MS and AD. In particular, AD has been known to increase Aβ and tau in CSF.^96^ Compared to healthy controls, Aβ levels were later found to be significantly reduced in MS patients.^97^ In contrast to these biomarkers, MS patients have been noted to have increased levels of CSF oligoclonal bands.^98^ There are several other clinically used biomarkers in the evaluation of MS including Anti-NZ, Nabs, IgG OB, IgG index, Anti-AQP4, Anti-JC virus, and Anti-VZV. Paul et al. (2019) previously compiled an array of biomarkers of MS and categorized them into exploratory, validated, and recently listed clinically useful biomarkers.^98^

The neuropeptide α-Calcitonin Gene-Related Peptide (α-CGRP) has been measured in CSF from patients with MS, AD, and healthy controls. MS patients did not have significantly different levels of α-CGRP from the controls. However, patients with AD had lower levels of α-CGRP compared to control and MS patient groups.^99^ Therefore, it may be possible that low α-CGRP could be used to aid in the diagnosis of AD in MS patients.

#### Blood-based miRNA evaluation using next-generation sequencing

An evaluation of a 12-miRNA signature containing miRNAs brain-miR-112, brain-miR-161, hsa-miR-5010-3p, hsa-miR-1285-5p, hsa-let-7d-3p, hsa-miR-26a-5p, and hsa-miR-151a-3p (which are all upregulated in patients with AD), and miRNAs hsa-miR-107, hsa-miR-535-5p, hsa-let-7f-5p, hsa-miR-103a-3p, and hsa-miR-532-5p (which are all downregulated in patients with AD).^100^ This evaluation provided a 93% accuracy, a 95% specificity, and a 92% sensitivity for the detection of AD. The utility of this tool in evaluating MS patients with symptoms of AD remains somewhat unclear. However, it may be possible that evaluation to determine the presence of these miRNA markers may indicate the development of AD in patients with a pre-existing diagnosis of MS.

#### Evaluation of biomarker presence in tears

A meta-analysis performed by Król-Grzymała et al. (2022) explored published results of differences in tear biomarkers between patients with MS, PD and AD.^101^ Biomarkers of AD included lipocalin-1, lysozyme-C, lacritin, amyloid proteins, t-Tau, and p-Tau. Biomarkers for MS included oligoclonal bands, free carnitine, lipids containing choline, acylcarnitines, and some amino acids. This study provided interesting data and new insights for improving clinical outcomes for patients with neurodegenerative diseases. Many of the proteins described in this study could be implemented clinically. However, the current understanding is insufficient to indicate reliable tear-related biomarkers for MS. In order to constitute a standard for early and non-invasive diagnosis of MS, this study should be performed on a much larger group of patients with neurodegenerative diseases. Regardless of this short-coming, presence of AD biomarkers in patients with an existing diagnosis of MS would provide supporting data for a diagnosis of AD.

#### Metabolomics

A potent method for studying diseases is to look for distinct metabolite signatures, which may serve as biomarkers. It may also be able to indicate whether disease-modifying therapies will be beneficial for treating MS.^102^ Unique metabolic signatures have been identified in several studies: the indicators include serum phospholipids, altered bile acid metabolism,^103,104^ abnormalities in aromatic amino acid metabolism,^105^ and pro-resolving lipid mediators in MS subjects compared to healthy subjects. Furthermore, a modified metabolite signature during a relapse of the disease^106,107^ may be developed as a metabolic biomarker for the advancement of MS disease.

In a similar vein, hundreds of distinct metabolites present in saliva have been investigated to determine which ones might be able to predict the presence of AD.^108^ This study demonstrated the ability of salivary metabolite markers to distinguish between patients with AD, presymptomatic (PP), and mild cognitive impairment (MCI). The markers hydroxylysine—H2O, glutamine-carnitine, and glucosyl galactosyl were used to identify AD and PP patients. Phenylalanyl-proline and alanyl-phenylalanine were the most effective in differentiating between the AD and MCI groups. Furthermore, they were able to differentiate AD from PP and MCI with good diagnostic performance by using positively confirmed metabolites,^108^ thereby allowing researchers to bring forward a metabolite assay that may be used to differentiate between patients with only MS and others with AD comorbidity.

The list above provides methods that have been recently described as possible ways to clinically evaluate the potential development of AD in patients with an existing diagnosis of MS. However, other areas of interest are still being researched, and future discoveries within the areas listed below may provide additional methods for evaluating the development of AD in patients with MS:

#### Homocysteine, vitamin B12 and folate

In vascular dementia and AD, moderately elevated Homocysteine (Hcy), vitamin B12, and folate have been found to be modifiable risk factors for cognitive impairment.^109^ Hcy-lowering treatment and improved cognition have produced mixed results.^110^ High levels of Hcy occur due to upregulated metabolism; whether vitamin B12 substitution could resolve potential imbalances in these pathways, and if they are causal or surrogate determinants of MS-related cognitive impairment, remains to be determined.^111,112^ Future research may determine if varying levels of homocysteine, vitamin B12, and folate may be able to elucidate isolated MS or AD pathogenesis from AD development in patients with a pre-existing diagnosis of MS.

#### Symbol digit modalities test (SDMT)

SDMT has previously been used to evaluate the onset and progression/severity of MS in the clinical setting.^5^ Previous use of SDMT testing included both cognitive (determination of answer) and movement (physical movement used to indicate the chosen answer) components. However, it has recently been suggested to remove the movement component of the examination and evoke oral responses from patients to distinguish delays caused by cognitive dysfunction versus motor dysfunction. By focusing on oral responses, the test deals with processing speed, working memory, paired-learning, and visual scanning. Some of these delays can be seen in patients with AD. However, research has not been conducted currently enough to determine if SDMT may be a viable tool for not just grading the severity of delays that may occur in AD, but also seeing if SDMT is able to distinguish between MS and AD. SDMT generally has a low specificity for MS, but determining any differences between AD and MS may allow for a new method of clinical evaluation.

#### Gut microbiome

The microbiome is a potentially important environmental factor that influences the pathophysiology of disease.^113^ Research has revealed that gut microbiota may impact not only the peripheral,^114^ CNS,^115^ and immune compartments, but also physiology and behavior. Since the gut microbiota can influence demyelination^116^ and BBB permeability,^117^ stool samples can serve as indicators of the gut microbiota and potentially help predict the risk of relapse in MS.^118,119^

The microbiome of MS patients has been observed to exhibit elevated levels of Pseudomonas, Mycoplasma, Haemophilus, Blautia, and Dorea genera. Conversely, the control group has demonstrated higher levels of Parabacteroides, Adlercreutzia, and Prevotella genera. Similarly, Saccharomyces and Aspergillus were found in higher concentrations in MS patients; the former was positively correlated with circulating basophils but negatively correlated with regulatory B cells, while the latter was positively correlated with activated CD16+ dendritic cells.^120^ Additionally, there appeared to be fewer butyrate products in the gut microbiota of MS patients.^121^ Butyrate can upregulate Treg populations via G-protein-coupled receptors for short-chain fatty acids (SCFAs) and enhance the synthesis of anti-inflammatory cytokines, including IL-10 and IL-4. This, in turn, promotes an anti-inflammatory state via the IL-10-mediated function of T cells and antigen-presenting cells.^122^

Furthermore, adjustments to the microbiota in AD patients were linked to elevated levels of pro-inflammatory cytokines in their unstimulated, non-centrifuged blood. Dysregulation of the microbiota and systemic inflammation may be linked to neurodegeneration in AD, as evidenced by the increased abundance of pro-inflammatory Escherichia and Shigella and the decreased population of anti-inflammatory Escherichia rectale, which may be associated with a peripheral inflammatory state in patients with cognitive impairment and brain amyloidosis.^123^ It is, however, crucial to remember that these findings are based on small studies. Large cohort studies, including longitudinal ones, are needed to fully evaluate the role played by the microbiota in the development of AD and its causal relationships. Further research comparing changes in gut microbiota between patients with only MS and patients with MS who later develop AD would provide another method of clinical evaluation.

### Study limitations

The current database searches identified a total of 101 cases in which a diagnosis of AD had been given after a diagnosis of MS. There are several limitations of this study:

The study by Cho et al. (2023) confirmed the diagnosis of MS & AD coexistence. However, authors failed to mention exact methods of diagnosis.^12^

There were 1548 new cases of comorbid MS and AD between all database searches including TriNetX. Adding the 24 cases identified by Luczynski et al. (2019), the total number of comorbid cases reached 1572.^1^ However, due to access limitations to patient records within the US, this number is likely not all-encompassing and could be a drastic underestimation. The rates of occurrence, however, may be accurate, depending on the demographic variations across the US. We are unable to confirm with certainty that cases within the TriNetX search were completely independent from those cases identified in PubMed, Clinical Key, BioMed Central, and Europe PubMed Central searches.

Except for criterion 3, i.e., not reporting the exact number of cases with coexisting MS and AD, the study by Mahmoudi et al. (2022) met other search criteria for this review.^124^ We, therefore, did not include this study in our review, however, the study made it clear that a significant number of patients could be diagnosed with MS-AD comorbidity. Compared to healthy controls, they determined that patients with MS between the ages 45-64, were seven times more likely to be clinically diagnosed with early AD. Furthermore, there was also an increase from 3.3% to 4% for late-onset AD beyond age 65 years.^124^

It is likely that most of the cases diagnosed with AD were not pathologically confirmed. Clinics without cognitive subspecialists, diagnostic labs and post-mortem confirmation may diagnose AD when in fact it is caused by other conditions. For example, without comprehensive evaluation and follow-up, the cognitive profile of later stage progressive MS can be mistakenly identified with AD. This issue is further exacerbated due to physicians’ hesitancy to attribute dementia to MS—given the history of underappreciation for cognitive decline in MS.

There was no consistency between diagnostic methods used for AD identification in MS patients across studies. Each used an array of techniques including, but not limited to, CSF biomarker analysis and Amyloid PET imaging to diagnose the presence of AD. The only way for a definitive diagnosis of AD is via autopsy. However, all reviewed articles suggest that each used a form of identification for AD that is scientifically supported and/or used clinically.

It is a possibility that there could be cases published outside of the databases that we searched in this review. Another possibility could be articles not found via a PubMed search before 2017. This is because the study by Luczynski et al. (2019) only considered cases found in PubMed before 2017, and the current study only searched four databases since 2018.^1^ Even with these limitations, we found a significant number of cases with MS-AD comorbidity.

Although other confounding factors likely present in respective populations, the development of AD in MS either introduces additional risk factors that may contribute to morbidity or accelerates the progression to morbidity in MS. Therefore, further research is needed to determine whether the MS-AD comorbidity has any impact on patient mortality.

### Conclusion

The goal of this review was to further clarify our understanding of MS-AD comorbidity in the aging MS population and to provide support for a mechanistic link between AD and MS. Given the growing body of literature showing connections between the glymphatic system and sleep in MS pathophysiology, we suggest that this relationship is further scrutinized in the future. For example, more precise glymphatic function measures such as contrast-based imaging and/or longitudinal studies should be utilized to better understand the dynamics of glymphatic-related damage in MS.

The review identified several clinically available measures that should be investigated in MS as risk markers for developing AD. Such measures can ensure that treatment can be initiated with maximal efficacy. For example, further research should be conducted to refine a methodical approach to evaluate MS patients’ cognitive as well as olfactory status—a potential diagnostic tool for AD in MS. Such an approach, however, may require costly initial evaluations for AD pathology either using amyloid PET imaging or CSF biomarkers.

The fact that etiological factors implicated in MS progression have the potential to be modified prior to disease onset is encouraging; can pave the way for potential preventive measures. Such measures, however, need to be tested in high-risk population groups, which is costly and time-consuming. In the meantime, early treatment options for MS patients who are at risk of developing cognitive impairment are desperately needed to minimize the morbidity associated with MS.

Vitamin D and B12 have been associated with cognitive performance.^35,125,126^ Longitudinal studies using the SDMT suggest that s25(OH)D might be related to information processing speed. These studies hypothesized neuroprotection as subserving the longitudinal causal associations between higher vitamin D levels and the preservation of information processing speed. However, vitamin D supplementation has not been effective in improving cognition.^35,90,125,127^ Some studies have associated vitamin D deficiency with worse performance on measures of spatial working memory and visual memory, but not verbal learning.^128,129^ Although inconclusive, vitamin substitution and dosing in MS must be further evaluated. For example, it may be useful to investigate s25(OH)D and B12-status in order to establish normal serum levels in MS patients; to ensure that assumed effects exceed possible risks for harm.

Regardless of the accuracy of exact numbers, it is clear that an abundance of cases exists with a diagnosis of both MS and AD. It is critical that clinical evaluation measures are clearly defined for improved diagnosis for AD in MS patients. By better understanding MS as a disease continuum, there is potential for early intervention. Insights provided by this review could facilitate early diagnosis of cognitive impairment risk in MS and therefore, better cognitive decline management during disease progression. Finally, this review highlighted ways to overcome common limitations in existing literature; to facilitate acquisition of reliable quantitative, not solely qualitative data for future research.
